# Pridopidine, a Potent and Selective Therapeutic Sigma-1 Receptor (S1R) Agonist for Treating Neurodegenerative Diseases

**DOI:** 10.3390/ph18121900

**Published:** 2025-12-17

**Authors:** Noga Gershoni Emek, Andrew M. Tan, Michal Geva, Andrea Fekete, Carmen Abate, Michael R. Hayden

**Affiliations:** 1 Prilenia Therapeutics, BV, 1411 DC Naarden, The Netherlandsmichal.geva@prilenia.com (M.G.); 2 Department of Neurology, School of Medicine, Yale University, New Haven, CT 06510, USA; 3 SigmaDrugs Research, 1012 Budapest, Hungary; 4 Department of Pharmacy–Pharmaceutical Sciences, University of Bari “Aldo Moro”, 70125 Bari, Italy; 5 Department of Medical Genetics, Centre for Molecular Medicine and Therapeutics, University of British Columbia, Vancouver, BC V5Z 4H4, Canada

**Keywords:** Sigma-1 receptor, S1R agonist, pridopidine, neuroprotection, neurodegenerative disease

## Abstract

Pridopidine is a highly selective sigma-1 receptor (S1R) agonist in clinical development for Huntington’s disease (HD) and amyotrophic lateral sclerosis (ALS). The S1R is a ubiquitous chaperone protein enriched in the central nervous system and regulates multiple pathways critical for neuronal cell function and survival, including cellular stress responses, mitochondrial function, calcium signaling, protein folding, and autophagy. S1R has a crucial role in the ER mitochondria-associated membrane (MAM), whose dysfunction is implicated in several neurodegenerative diseases. By activating the S1R, pridopidine corrects multiple cellular pathways necessary to the cell’s ability to respond to stress, which are disrupted in neurodegenerative diseases. Pridopidine restores MAM integrity; rescues Ca^2+^ homeostasis and autophagy; mitigates ER stress, mitochondrial dysfunction, and oxidative damage; and enhances brain-derived neurotrophic factor (BDNF) axonal transport and secretion, synaptic plasticity, and dendritic spine density. Pridopidine demonstrates neuroprotective effects in in vivo models of neurodegenerative diseases (NDDs). Importantly, pridopidine demonstrates the biphasic dose response characteristic of S1R agonists. In clinical trials in HD and ALS, pridopidine has shown benefits across multiple endpoints. Pridopidine’s mechanism of action, modulating core cellular survival pathways, positions it as a promising candidate for disease modification for different nervous system disorders. Its broad therapeutic potential includes neurodevelopmental disorders, and rare diseases including Wolfram syndrome, Rett syndrome, and Vanishing White Matter Disease. Here, we review the experimental data demonstrating pridopidine’s S1R-mediated neuroprotective effects. These findings underscore the therapeutic relevance of S1R activation and support further investigation of pridopidine for the treatment of different neurodegenerative diseases including ALS and HD.

## 1. The Sigma-1 Receptor (S1R)

The Sigma-1 Receptor (S1R) is a small (25 kDa), highly conserved transmembrane protein that is ubiquitously expressed throughout the body and is especially enriched in neurons and glia in the central nervous system (CNS). Despite its name, no direct transduction pathways have been associated with S1R activity and its signaling [1]. Rather, the S1R exerts its effects by binding and modulating a plethora of protein partners [2,3].

Accumulating evidence from the past two decades demonstrates that the S1R is essential for maintaining cellular health and proper function. S1R function can be modulated by agonists and antagonists, which differentially affect both its oligomerization and binding partners, yielding distinct downstream effects [2,4].

**Figure 1:**
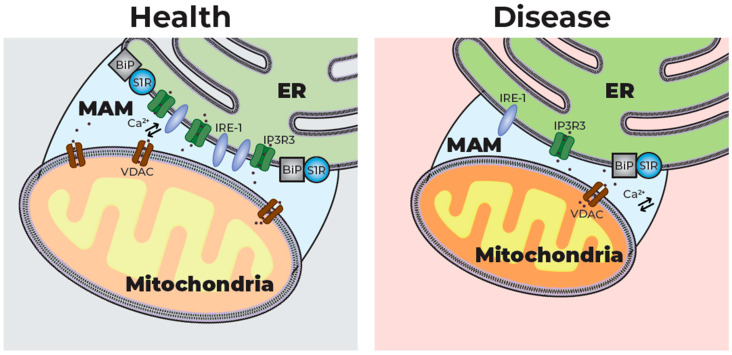
The MAM in health and disease: The mitochondria-associated membrane (MAM) is a specialized subdomain of the endoplasmic reticulum (ER) that forms close contacts with mitochondrial outer membranes, enabling the bidirectional exchange of ions, lipids, and signaling molecules. In healthy cells, the MAM plays a crucial role in tethering the ER to the mitochondria and facilitating the transport of ions, lipids, and signaling molecules between the two compartments. In disease states such as neurodegeneration, MAM integrity is disrupted, causing a reduction in ER–mitochondria tethering and signaling, contributing to oxidative and ER stress and cell death. BiP—binding immunoglobulin protein; S1R—Sigma-1 receptor; VDAC—voltage-dependent anion channel; IRE-1—inositol-requiring enzyme 1; IP_3_R3—inositol 1,4,5-triphosphate receptor type 3.

The S1R is primarily localized to the endoplasmic reticulum (ER) membrane, but it may be able to translocate between the ER, mitochondria and plasma membrane [5]. The S1R forms microdomains within the ER membrane, and is preferentially localized in proximity to lipid droplets and at inter-organelle contact sites. It is particularly enriched at the ER mitochondria-associated membrane (MAM). The MAM has a unique composition that differentiates it from the rest of the ER membrane. It contains higher levels of cholesterol and ceramides, as well as enzymes necessary to facilitate its function as a specialized ER signaling domain [6] (Figure 1).

By regulating signaling between the ER and the mitochondria, the MAM orchestrates multiple cellular functions, including lipid exchange, mitochondrial DNA exchange, Ca^2+^ signaling, autophagy, and inflammasome creation [7,8]. Structural and functional dysfunction of the MAM is associated with numerous neurodegenerative diseases, as it coordinates the various cellular processes commonly disrupted in these diseases [9,10]. For example, both TDP-43 and FUS, proteins that play key roles in ALS pathology, can alter MAM tethering and disrupt Ca^2+^ homeostasis [11,12]. Similarly, the Parkinson’s Disease (PD)-associated protein Parkin plays a protective role by influencing MAM structure and regulating Ca^2+^ signaling, which is lost when the protein is mutated [10,13]. In Alzheimer’s Disease (AD), abnormal MAM function and ER–mitochondria cross-talk are suggested to be early pathophysiological changes, affected by amyloid-β [10,14]. Conversely, in HD, excessive mitochondrial fission disrupts MAM integrity [9].

The S1R is key to maintaining MAM integrity [7]. Under basal conditions, the S1R is tethered to the ER protein binding immunoglobulin protein (BiP). Upon ER Ca^2+^ depletion or agonist binding, it dissociates from BiP to interact with and regulate the function of other proteins throughout the cell, thereby enhancing a variety of cellular functions leading to cellular survival [5,7,15].

A primary function of the S1R is the regulation of Ca^2+^ signaling between the ER and mitochondria, which it does by binding to and stabilizing the inositol 1,4,5-trisphosphate receptors (IP_3_Rs), which are the main pathway for Ca^2+^ transfer from ER stores to the mitochondria [8,15,16]. Another mechanism by which the S1R mitigates ER stress is by binding to and stabilizing the ER stress sensor inositol-requiring enzyme-1 (IRE1), one of the three arms of the unfolded protein response (UPR) that is particularly sensitive to mitochondria-derived reactive oxygen species (ROS) [17,18,19]. Additional cellular pathways activated by S1R modulation include the attenuation of ROS, mitochondrial function, and transcriptional regulation [1,20], all of which facilitate pro-survival mechanisms. These pathways are discussed in greater detail below.

Thus, the S1R can be viewed as a molecular switch influencing cell death or survival in response to cellular stress.

## 2. Neurodegenerative Diseases

Today, more than 65 million people worldwide are affected by neurodegenerative diseases, including AD, PD, amyotrophic lateral sclerosis (ALS), Huntington’s disease (HD), retinal degeneration, and others, posing a challenge and a burden to society [21]. The prevalence of neurodegenerative diseases is increasing with the aging population, which increases the risk of individuals developing these diseases, together with environmental factors and lifestyle changes [22,23].

These debilitating diseases differ significantly in their underlying etiology, affecting specific cell types in different regions of the brain, which influence the wide range of clinical presentations [24,25]. However, the shared progressive nature of these diseases results from common pathophysiological cellular mechanisms [23,26] and pathways, including ER stress, mitochondrial dysfunction, protein aggregation and decreased autophagy, impaired cellular transport, neurotrophin dysfunction, and neuroinflammation [25].

Another shared aspect of neurodegenerative diseases is the limited treatment options [27]. This is especially true in the case of rare diseases, such as HD, where patients are limited to symptomatic or supportive care, and ALS, where, despite recent advances in treatment options, the unmet need remains urgent [28,29].

HD is a rare, lethal, and inherited neurodegenerative disease, leading to progressive neuronal death and manifesting as a decline in cognitive, behavioral, and motor functions [30,31]. HD arises from an abnormal repeat amplification of the CAG trinucleotide of the *Htt* gene, resulting in the production of misfolded, mutant Htt protein (mHtt), which creates aggregates in neurons, a hallmark of the disease. mHtt induces neurodegeneration through toxic gain-of-function mechanisms together with some loss of function of wild-type Htt [32]. The pathological effects of mHtt include early transcriptional dysregulation, synaptic dysfunction, altered axonal trafficking, impairment of protein homeostasis, impaired autophagy, oxidative damage, mitochondrial dysfunction, and extra-synaptic excitotoxicity [33]. A direct effect of the mutated Htt protein is the transcriptional downregulation of the brain-derived neurotrophic factor (BDNF), which is essential for the development, maintenance, and survival of neurons [34]. The progressive neurodegeneration and death initially of these neurons in the caudate and putamen, followed by more widespread cell death in the cortex, results in the varying clinical manifestations of the disease [35,36].

ALS is a progressive, fatal neurodegenerative disorder characterized by the degeneration and eventual death of motor neurons, leading to muscle weakness, paralysis, and ultimately death from respiratory failure. The causes of ALS are multifactorial, determined by a complex combination of genetic, epigenetic, and environmental factors. Up to 10% of cases have a family history of ALS [37]. Despite this etiological difference, the pathophysiology of ALS is similar to that of HD and other neurodegenerative diseases, and includes oxidative stress, mitochondrial dysfunction, protein aggregation, and impaired axonal transport [38].

Recent years have seen significant advances in the understanding of neurodegenerative diseases and their underlying biology, driving the development of novel therapeutics. Among these, the S1R has emerged as a promising therapeutic target, and currently, both novel investigative and repurposed drugs targeting the S1R are being explored for their effect on diseases such as AD, PD, HD, and ALS [5,39,40,41].

Mutations in the S1R can be rare causes of ALS. Mutations in the S1R gene demonstrate a gene-dose relationship with disease severity [42]. For example, complete loss-of-function mutations, resulting from frameshift mutations or null alleles, is causative for a more severe form of ALS with juvenile onset [43,44]; partial loss-of-function missense mutations result in adult-onset or slower progression compared to null alleles [45,46,47]. Additionally, mutations in different regions of the S1R gene are associated with distal hereditary motor neuropathy (dHMN), a heterogenous group of diseases with common features of progressive muscle wasting without sensory loss [48,49,50,51,52].

Polymorphisms in the S1R gene (Q2P and C240T/G241T) may affect the risk of developing AD in APOE e4 carriers [53,54]. Moreover, a decrease in S1R expression levels may contribute to AD pathology, as a possible result of DNA polymorphisms in the S1R gene (C240T/G241T) [55,56].

S1R dysfunction may also contribute to the progressive nature of neurodegenerative diseases. In animal models, genetic deletion of the S1R results in increased ER stress, reduced proteosomal activity, increased oxidative stress, and increased toxic aggregation, all of which contribute to the degenerative process in neurons [57,58]. SOD1^G93A^ ALS model mice in which the S1R was genetically silenced, demonstrated earlier weight loss, faster disease progression, and reduced survival time [59]. Thus, S1R dysfunction exacerbates neurodegenerative mechanisms, leading to earlier or more severe phenotypes. The potential clinical applications of S1R agonism in NDDs are further elaborated in Section 7.

## 3. Pridopidine

Pridopidine is a small molecule in late-stage clinical development for HD and ALS. It is a highly selective and potent S1R agonist (Ki = 57 nM), with low affinity for other CNS receptors, such as the adrenergic α2C (ADRα2, Ki = 1580 nM), dopamine D3 (Ki = 1630 nM), Sigma 2R (Ki = 5450 nM), and dopamine D2 (Ki = 2950 nM) receptors [60].

Pridopidine is the most selective S1R agonist in clinical development [5,60]. Pridopidine shows a 28-fold higher affinity for the S1R vs. the adrenergic a2C and a 96-fold higher affinity vs. the S2R [60]. On the other hand, blarcamesine, a non-selective S1R agonist in clinical development for AD [61], has moderate affinity for the S1R (Ki = 3700 nM) with the highest affinity for the muscarinic M1 receptor (Ki = 500 nM) [62], showing 0.14-fold lower selectivity for the S1R. Dextromethorphan, an S1R agonist and NMDAR antagonist, is a commonly used cough suppressant, has comparable affinities for the S1R (~100 nM) and the NMDA-R (~500 nM, ~5-fold higher affinity for the S1R) [63]. Donepezil is also a non-selective S1R agonist with affinity for the AChEi (5–7 nM) and lower affinity for the S1R (14.6 nM, 0.4-fold selectivity for the S1R) [63,64]. The antipsychotics chlorpromazine and haloperidol are S1R antagonists with moderate and high affinity for the S1R (Ki = 180 and 3.7 nM, respectively) [65,66]. However, both demonstrate much higher affinity for dopamine receptors, chlorpromazine for the D1R (Ki = 56 nM, ~0.03-fold lower than for the S1R) and haloperidol for the D2R (Ki = 0.89 nM, ~0.24-fold lower than for the S1R) [67,68,69]. Similarly, other drugs such as SSRI inhibitors, including imipramine, fluoxetine, citalopram, and fluvoxamine, show the highest affinity for the SERT, while showing low S1R affinities (<0.05-fold selectivity for the S1R vs. SERT) [66] (see Table 1).

Pridopidine rapidly traverses the blood–brain-barrier and demonstrates selective and robust target engagement in the human brain [70,71]. At the recommended clinical dose of 45 mg bid, pridopidine achieves over 90% occupancy of the S1R in the human brain, compared to negligible binding at the dopamine D2/D3 receptors [70]. Pridopidine binds to a hydrophobic binding pocket at the C-terminal ligand-binding domain that resides in the ER lumen [72].

Numerous preclinical studies have evaluated the effects of pridopidine on crucial cellular mechanisms and cell survival, in multiple models of neurodegenerative disease. These studies, the majority of them in HD models, provide insight into its mechanism of action as an S1R agonist, as well as compelling evidence supporting its clinical development [60,73,74,75,76,77,78,79,80,81,82,83,84,85,86,87].

## 4. Mechanism of Action of Pridopidine

### 4.1. Preservation of MAM Integrity

MAM structure and function are disrupted in HD, leading to aberrant Ca^2+^ homeostasis, elevated levels of ER stress, and impaired mitochondrial function [88]. In striatal neurons derived from the YAC128 HD mouse model, the number of ER–mitochondria contact sites is significantly decreased compared to wild-type neurons, as is the percentage of mitochondrial surface covered by the ER. Colocalization of the S1R and inositol 1,4,5-triphosphate receptor (IP_3_R) with the mitochondria is also reduced, suggesting a decrease in tethering at the MAM. Pridopidine treatment (1 µM) rescued MAM integrity by restoring colocalization of S1R and IP_3_R with mitochondria and increasing the number of ER–mitochondria contact sites and the percentage of mitochondrial surface in contact with ER [81]. These data suggest that pridopidine preserves MAM integrity, maintaining Ca^2+^ flux and mitochondrial function. The restoration of Ca^2+^ homeostasis is a well-known effect of S1R agonism [16].

Striatal medium spiny neurons from YAC128 HD mice have significantly lower ER Ca^2+^ levels compared to WT neurons, which are restored by pridopidine treatment (100 nM and 1 µM). Importantly, this effect is exclusively mediated by the S1R, evidenced by the absence of a pridopidine effect in YAC128 neurons in which the S1R was genetically deleted [82]. Conversely, hippocampal neurons from AD mouse models in which presenilin-1 was deleted or a mutant version (M146V) was introduced have high, toxic ER Ca^2+^ levels. These elevated ER Ca^2+^ levels suppress neuronal store-operated calcium entry, which causes the destabilization of mushroom spines, detrimental to neuronal function. Pridopidine treatment (1 µM) restores aberrant Ca^2+^ levels in an S1R-dependent manner [83].

### 4.2. Regulation of ER Stress

Impaired Ca^2+^ homeostasis in the ER leads to ER stress, inhibiting the ER’s functionality in properly synthesizing and folding proteins. Thus, prolonged ER stress results in the accumulation of toxic proteins, a hallmark of neurodegenerative diseases that contribute to neuronal degeneration and subsequent death [89].

STHdh^Q7/7^ murine striatal cells that were transfected with the human *huntingtin* gene expressing either 20 (wild-type) or 96 (mutant) CAG repeats show intracellular aggregates of GFP-tagged asialoglycoprotein receptor H2a, a model misfolded secretory protein whose accumulation is an early indicator of ER stress. Pridopidine treatment (0.03–3 µM) reduced H2a aggregation in HD cells by 40% [84].

**Figure 2:**
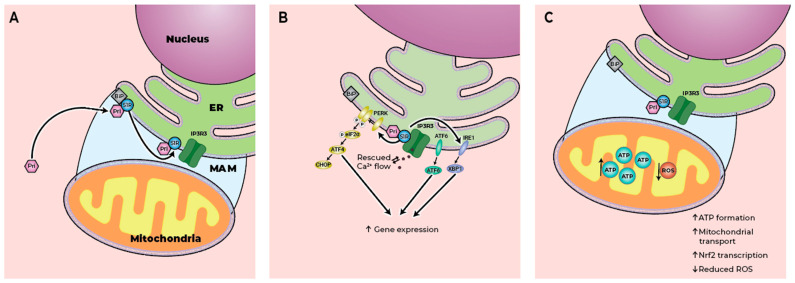
S1R activation by pridopidine alleviates ER and mitochondrial stress. (**A**)—Pridopidine binding to the S1R leads to its activation and dissociation from the ER protein BiP. Once free, the S1R can bind to various proteins, regulating their activity. (**B**)—Upon activation by pridopidine, the S1R stabilizes IP_3_ receptors at the ER–mitochondria interface, regulating calcium flow. Sustained S1R activation enhances signaling across all three branches of the UPR—PERK, IRE1α, and ATF6—leading to the upregulation of pro-survival pathways. (**C**)—S1R activation restores mitochondrial membrane potential, increasing mitochondrial respiration and ATP production, and reducing levels of ROS. ER—endoplasmic reticulum; Pri—pridopidine; BiP—binding immunoglobulin protein; S1R—Sigma-1 receptor; IP_3_R3—inositol 1,4,5-triphosphate receptor type 3, PERK—protein kinase RNA-like ER kinases; eIF2—eukaryotic initiation factor 2; ATF4—activating transcription factor 4; CHOP—C/EBP homologous protein; ATP—adenosine triphosphate; ROS—reactive oxygen species.

ER stress upregulates the unfolded protein response (UPR), a three-arm process activated to counteract ER stress. Phosphorylation of the eukaryotic translation initiation factor 2a (eIF2a) is a central event in the UPR process. The aggregation process of mHtt induces all three branches of the UPR, with the most pronounced effect occurring on the PERK branch, which leads to the upregulation of UPR markers, including C/EBP homologous protein (CHOP) and growth arrest and DNA damage (GADD)34. Pridopidine treatment (0.3 and 3 µM) reduced the phosphorylation levels of eIF2a, as well as the protein levels of CHOP and GADD34. Moderate effects were also observed on the other two branches of the UPR, resulting in downregulated levels of ATF6 and XBP1s [84] (Figure 2).

These data emphasize that pridopidine significantly inhibits ER stress with a direct effect on the UPR and on the aggregation of misfolded proteins.

### 4.3. Modulation of Mitochondrial Function

Mitochondrial dysfunction and oxidative stress are early characteristics of neurodegenerative diseases [90]. In YAC128 HD neurons, the mitochondrial membrane potential is disrupted, affecting mitochondrial respiration and ATP production. These impairments make HD neurons more sensitive to oxidative stress.

Pre-treatment with pridopidine (0.1 and 1 µM) demonstrated beneficial effects on mitochondrial function in multiple models of HD, including cortical neurons, striatal neurons, and corticostriatal co-cultures from WT and YAC128 HD mice; in human induced pluripotent stem cell (iPSC)-derived neural stem cells (NSCs) from a heterozygous HD patient (HD-NSCs) with a normal allele of 19 CAG repeats and an expanded allele retaining 72 CAG repeats; and in lymphoblasts from human controls and HD patients (at 5 µM) [81].

Mitochondrial membrane potential is essential for cellular energy production as it directly affects ATP production. Both YAC128 HD cortical and striatal neurons exhibit lower mitochondrial membrane potential in response to mild induced mitochondrial depolarization, which is rescued by pre-treatment with pridopidine (0.1 and 1 µM). This protective effect was also observed in human HD lymphoblasts following a more potent oxidative stimulus of H_2_O_2_, where treatment with pridopidine (5 µM) 24 h prior to oxidative challenge showed the complete rescue of membrane potential. Importantly, this effect was completely abolished by the genetic silencing of the S1R [81].

The effect of pridopidine (1 µM) on mitochondrial respiration was evaluated in two cellular models: YAC128 cortical/striatal neurons and human HD-NSCs. In YAC128 HD neurons, both basal and maximal respiration are reduced compared to wild-type. Pridopidine significantly increased maximal respiration in both wild-type and HD neurons, as well as increasing basal respiration in wild-type neurons. In HD-NSCs, pridopidine (1 µM) significantly rescued both basal and maximal respiration. In both models, pridopidine (1 µM) rescued ATP production [81].

YAC128 HD neurons exhibit increased susceptibility to oxidative challenge, as demonstrated by a two-fold increase in mitochondrial-driven H_2_O_2_ levels (ROS) upon stimulation with antimycin A, an inhibitor of complex III of the electron transport chain. Pre-treatment with pridopidine (1 µM) completely abolished the elevation of ROS in response to this stimulus [81].

The S1R modulation of the cellular response to oxidative stress is mediated via the transcription factor Nrf2. In wild-type lymphoblasts, Nrf2 protein levels increase in response to oxidative challenge, an effect not seen in HD lymphoblasts. This suggests the impaired expression of the downstream antioxidant response element (ARE) genes, and indeed, two genes regulated by the NRf2-ARE pathway, Nqo1 and Hmox1, show reduced mRNA levels in HD lymphoblasts. The mRNA levels of both genes are increased in response to pridopidine treatment (5 µM), indicating that pridopidine’s rescue of the oxidative stress response involves the regulation of gene expression [80,81] (Figure 2).

Pridopidine induces transcriptional changes in multiple brain regions in HD model mice. YAC128 HD mice demonstrate gene expression changes starting from a pre-symptomatic stage, most notably in the striatum but also in the hippocampus and cortex. Accordingly, the effect of pridopidine (10 and 30 mg/kg, administered by oral gavage) on gene expression is also observed primarily in the striatum. Importantly, the transcriptional footprint of pridopidine demonstrates a reversal of the disease-specific expression observed in the YAC128 HD mouse model [77,80]. These include critical neuroprotective pathways such as brain-derived neurotrophic factor (BDNF), dopamine-1 receptor (D1R), and the glucocorticoid and calcium signaling pathways [80].

### 4.4. Autophagy Enhancement

Autophagy is an important biological process, essential for the removal of damaged organelles and toxic or aggregated proteins. In many neurodegenerative diseases, autophagy is disrupted, contributing to the impaired clearance and aggregation of neuronal proteins, which leads to the toxic aggregation of proteins, a hallmark of neurodegenerative diseases [91].

Loss-of-function mutations in the S1R, associated with ALS, have been shown to disrupt autophagy [92,93]. Activation of the S1R with PRE-084 or ANAVEX2-73 (blarcamesine) induces autophagy, increasing autophagic flux and reducing protein aggregation [94], supporting its potential as a therapeutic target for modulating autophagy.

An initial, essential step in initiating autophagy is the translocation of the transcription factor EB (TFEB) from the cytoplasm to the nucleus, where it induces the expression of key genes in the autophagy–lysosomal pathway [95]. Nucleocytoplasmic transport is facilitated by the nuclear pore complex (NPC), a large protein complex that ensures selective and efficient transport between the two compartments. The destabilization of the NPC as a result of alterations in the nuclear pore proteins (NUPs) diminishes transport efficiency [96,97,98]. In line with this, TFEB levels as well as its transcriptional targets are reduced in models of neurodegenerative diseases [99,100,101,102].

The S1R plays a key role in the stabilization of the NPC, and its overexpression has been shown to rescue deficits in nucleocytoplasmic transport in a drosophila model of ALS/FTD [103]. Activation of the S1R releases the S1R from BiP to act as a chaperone and stabilize POM121 [104]. POM121 is a key nucleopore protein (NUP) that acts as a gate-keeper, ensuring the stability of other NUPs at the NPC [86,105]. Its expression is reduced by the G_4_C_2_ hexanucleotide repeat elements (HRE) in the ALS-causative *C9orf72* gene, impacting the expression of seven additional NUPs and interfering with nucleocytoplasmic transport [105]. In the NSC-34 motor neuron-like cell line expressing a (G_4_C_2_)_31_ HRE, nuclear TFEB levels are significantly reduced, and autophagy is impaired, as evidenced by a decrease in the autophagy marker LC3-II. Both of these abnormalities are rescued by pridopidine treatment (1 µM) [86].

### 4.5. Increasing Brain-Derived Neurotrophic Factor (BDNF) Availability

BDNF is a key neurotrophic factor, essential for neuronal function, connectivity and survival. It is essential for corticostriatal connectivity, as it supports the formation, maintenance, and plasticity of synapses between cortical and striatal neurons [106]. BDNF in the adult striatum is primarily synthesized in the cortex, and relies on proper anterograde transport and secretion from cortical neurons as well as effective binding and downstream signaling by the TrkB receptor at the post-synapse [106].

BDNF plays a central role in HD. BDNF mRNA and protein levels are decreased in the brains of HD model animals, as well as in post-mortem brain tissue from HD patients, as a result of the mutant Htt protein [34,107]. Thus, a treatment that can enhance BDNF levels presents an intriguing opportunity for the treatment of HD. The overexpression of BDNF in the forebrain of YAC128 HD mice increased BDNF levels in the striatum, rescuing the abnormal spine phenotype of medium spiny neurons and preventing the loss of striatal neurons and brain atrophy. This resulted in the amelioration of motor dysfunction and reversal of cognitive deficits in these mice [108,109].

Pridopidine’s effect on BDNF secretion was demonstrated in B104 rat neuroblastoma cells, in which it enhanced secretion through an S1R-mediated mechanism [78]. This finding established BDNF secretion as a readout of S1R activation. But pridopidine’s effects on BDNF signaling are far more profound, affecting presynaptic dynamics, synaptic morphology, and post-synaptic signaling. These were evaluated using a microfluidic device “disease-on-chip” platform, in which primary neurons from the HD mouse model HdhCAG^140/+^ were used to reconstitute a functional corticostriatal network. All aspects of BDNF transport were reduced relative to wild-type neurons, in line with mHtt’s known function in axonal transport [107,110,111].

Within the presynaptic compartment, pridopidine (1 µM) improved all aspects of BDNF axonal transport, including global and anterograde velocity, the number of motile vesicles, and linear flow rate (a value composed of the velocity and number of motile vesicles providing a numerical estimate of overall transport), back to wild-type levels [87].

At the synapse, glutamate transmission as well as synaptic homeostasis are impaired in CAG140 neurons [110]. Treatment with pridopidine (1 µM) enhanced glutamate release long-term, suggesting maintenance of the synaptic transmission mediated by a presynaptic mechanism. Pridopidine treatment restored synaptic homeostasis, as revealed by synaptic markers spinophilin, VGLUT1, and PSD95 [87].

At the post-synapse, BDNF binds to the TrkB receptor, leading to its internalization and trafficking within the dendrite to induce pro-survival signaling. One of the pathways activated by TrkB is the MAP/ERK pathway, in which ERK is phosphorylated, activating regulators of protein translation, promoting anti-apoptotic gene expression, and inhibiting pro-apoptotic factors [106]. Phospho-ERK (pERK) signaling is downregulated in the HD striatum [110]. In dendrites of CAG140 striatal neurons, the global velocity of TrkB vesicles was reduced, while the number of static vesicles increased. Pridopidine treatment (1 µM) completely restored trafficking deficiencies, as well as restoring pERK to wild-type values [87].

**Figure 3:**
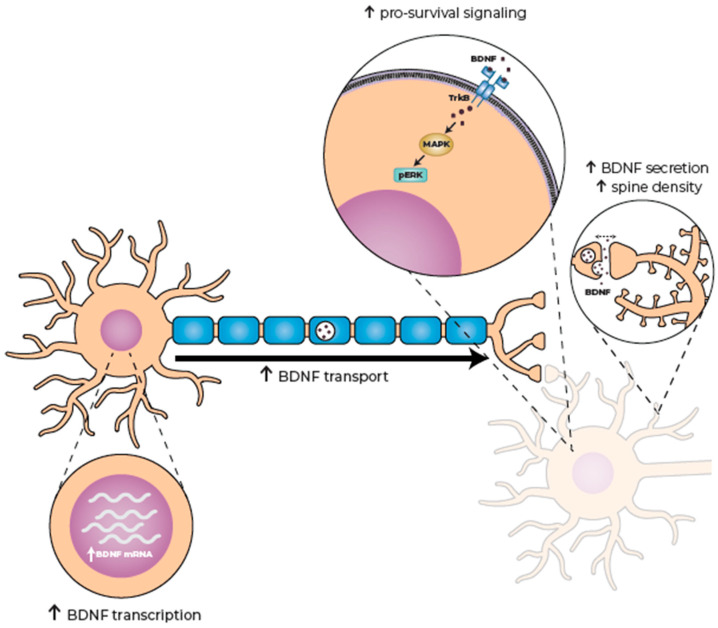
Pridopidine enhances BDNF production and signaling. Pridopidine activation of the S1R increases BDNF transcription, axonal transport, and synaptic release. At the post-synapse, BDNF signaling is enhanced via the TrkB receptor, leading to increased pro-survival signaling and regulating dendritic spine density. BDNF—brain-derived neurotrophic factor; TrkB—tropomyosin receptor kinase B; MAPK—mitogen activated protein kinase; pERK—phosphorylated extracellular signal-regulated kinase.

Similarly to in HD neurons, the axonal transport of BDNF is disrupted in motor neurons derived from the mSOD1^G93A^ mouse model of ALS, with reductions both in the instantaneous velocities of transporting vesicles and the number of stops along the way. Pridopidine treatment (0.1 and 1 µM) rescued both of these abnormalities. In mSOD1 cultures, pridopidine increased phosphorylation of ERK downstream to TrkB activation by BDNF. A similar upregulation of BDNF, together with an increase in ERK phosphorylation, was observed in striatal neurons from the 6-OHDA mouse model of PD, treated with pridopidine (0.3 mg/kg) [76]. These data show that pridopidine’s effects on BDNF are not limited to HD neurons (Figure 3).

### 4.6. Preservation of Dendritic Spine Density

Dendritic spines are microscopic-sized post-synaptic structures that compartmentalize the cellular machinery underlying excitatory synaptic function in the CNS. These are highly dynamic structures that can grow, change shape, or retract in response to neuronal activity, providing the framework for plasticity in the brain’s connectivity and circuitry, which underlie processes of learning and memory [112,113,114]. An enhanced spine density mediates greater neuronal connectivity.

In HD, perturbed corticostriatal synaptic function and connectivity are an early neuropathological feature, possibly resulting from the destabilization of dendritic spines. The dendritic spine density is significantly decreased compared to that in wild-type neurons. In the YAC128 HD Mouse model, medium spiny neurons (MSNs) display age-dependent spine loss compared to wild-type MSNs [115]. Pridopidine treatment (1 µM) completely prevented spine loss [82] (Figure 3).

Pridopidine (1 µM) similarly prevented the loss of mushroom spines in hippocampal neurons from models of AD, in which toxicity was induced by Aβ_42_ or H_2_O_2_ toxicity or genetic knock-in models of presenilin (PS1-KI) and amyloid precursor protein (APP-KI). Long-term potentiation (LTP) in hippocampal neurons is impaired by Aβ_42,_ and fully restored by pridopidine treatment (30 and 100 nM) [74,83].

Homeostatic plasticity (HSP) refers to the mechanisms that maintain the stability of neuronal networks in the face of ongoing changes, underlying development, learning, cognitive flexibility, and pathological insults. Intriguingly, the set of genes involved in HSP overlaps those involved in neurodegenerative diseases, including HD and ALS [116].

BDNF and calcium signaling are both points of overlap in HSP and HD. BDNF is released in an activity-dependent manner, and its activity via the TrkB receptor is a signal for the up- or down-scaling of synapses [117]. Disrupted BDNF-TrkB signaling, which is observed in HD, interferes with these processes. Similarly, disrupted Ca^2+^ homeostasis, which is a feature of HD and other neurodegenerative diseases, can also interfere with HSP, as Ca^2+^ signaling is crucially involved in HSP [117,118].

When wild-type neurons are treated with tetrodotoxin (TTX) to silence action potentials, an increase in both the amplitude and frequency of miniature excitatory postsynaptic currents is recorded, indicating functional HSP. YAC128 HS neurons, on the other hand, show no response to action potential silencing—indicating impaired HSP. Dysregulated HSP is completely rescued by pridopidine treatment (1 µM) [85].

Taken together, these data demonstrate that, by enhancing BDNF axonal transport and secretion and regulation of Ca^2+^ homeostasis, pridopidine restores the ability of HD cortical neurons to modulate BDNF release, which is crucial for synaptic plasticity.

### 4.7. Restoration of Neuromuscular Junction Activity

Pridopidine’s effects on the cellular pathology of ALS were studied in a simplified motor unit system, which co-cultured spinal cord explants and primary myocytes in microfluidic chambers, and in which functional neuromuscular junctions (NMJs) are established. In cultures derived from mSOD^G93A^ mice, the number of active NMJs is significantly decreased compared to wild-type controls. Pridopidine treatment (0.1 µM) restored NMJ activity and alleviated the cell death observed in mSOD1 cultures [79].

### 4.8. Enhancement of Neuroprotection

Pridopidine demonstrates neuroprotection against cell death in both HD and ALS models [73,86]. Cortical and striatal neurons transfected with mHtt (82 CAG repeats in the exon 1 of the huntingtin gene, called 82Q) show more than two-fold greater cell death than neurons transfected with wild-type Htt (20Q), which is completely reversed by pridopidine treatment (1 µM) [73]. A similar effect is also observed in HD patient-derived induced pluripotent stem cells (iPSCs) [73].

**Figure 4:**
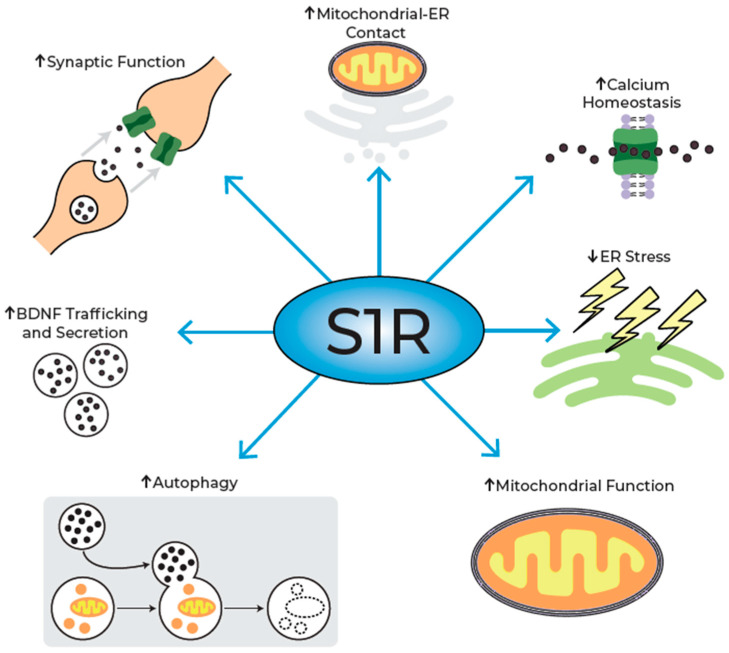
The multiple effects of S1R activation by pridopidine. Pridopidine activation of the S1R results in an increase in Mitochondrial–ER contacts, facilitating calcium homeostasis. This regulates ER stress and enhances mitochondrial function, as well as regulates autophagic processes. S1R activation also increases BDNF trafficking and secretion, leading to enhanced synaptic function. All of these processes are disrupted in neurodegenerative diseases, and their activation is neuroprotective.

Similarly, pridopidine significantly delays the death of cultured motor neurons (MNs) from the ALS SOD1 mouse model (SOD1^G93A^). The observed neuroprotection is a result of the pridopidine’s modulation of the numerous cellular processes detailed above (Figure 4).

## 5. The Biphasic Effect of S1R Activation

Notably, pridopidine exhibits a biphasic dose response, which is an intrinsic characteristic of S1R activation, in which an optimal effect is observed at mid-range concentrations but diminished at higher and lower doses. Pridopidine’s effects were evaluated in various disease model systems, with the majority of effects observed at optimal efficiency within the 0.1–5 µM range. Depending on the model used, at low (<100 nM) and high (>5 µM) doses, the effects were diminished [78,84,87]. Numerous studies have documented the biphasic effect of S1R activation in preclinical, in vitro, and in vivo models, as well as in clinical trials [1,16,119,120,121,122].

The underlying mechanisms of the biphasic response are under ongoing investigation, but are thought to involve multiple factors, including signaling pathways, stress response mechanisms, and interactions with various ligands.

Several pharmacological characteristics of the S1R may contribute to the effect. For example, changes in the S1R oligomerization state may contribute. The S1R is present in both monomeric and oligomeric states, and the transition between these states is altered by ligand binding, affecting the functional activity of the S1R [1,123,124,125].

The cellular dynamics of the S1R may also play a part in the biphasic response. The S1R creates distinct lipid-enriched microdomains within the ER membrane. The activation of the S1R results in its translocation from the ER membrane to other cellular compartments, as well as changes in its expression levels. The levels of S1R and its cellular localization can also affect its activity [1,15,126,127].

An additional aspect that highly influences this biphasic response is selectivity for the S1R over the Sigma-2 receptor (S2R). The S2R is an ER transmembrane protein as well. However, it is not structurally related to the S1R, yet shares similarities in pharmacological profile and ligand binding [128,129]. Intriguingly, activation of the S1R and S2R appears to have opposing effects. For example, while S1R activation promotes neuroprotection, S2R agonists promote neurodegeneration and cell death, making them potential anti-cancer drugs [130,131,132]. Thus, an S1R agonist with low selectivity for the S2R may have a narrow therapeutic window and activate the S2R at similar therapeutic doses, thus decreasing the neuroprotective effects of S1R activation [1,133,134,135,136,137].

Contrastingly, S2R antagonists improve lysosomal function and intracellular trafficking, which may improve neuronal survival [88,138,139,140,141]. Indeed, the S2R antagonist CT1218 is in clinical development for the treatment of AD, having shown potential disease-modifying effects [142,143,144,145]. The combination of pridopidine with the S2R antagonist FA10 resulted in a beneficial effect, alleviating mHtt-induced neuronal toxicity, that was superior to the effect of each compound when administered individually, suggesting an additive effect. This presents an interesting possibility for combination therapy.

## 6. Beneficial Effects of Pridopidine in Models of Neurodegenerative Diseases

Pridopidine’s neuroprotective effects on neuronal survival and function are translated to in vivo effects in the YAC128 mouse model. These HD mice recapitulate many of the signs of HD, including disturbed motor function, impaired learning abilities, and psychiatric abnormalities such as anxiety and depression. Mice from early, pre-symptomatic stages of the disease (1.5 months of age) were treated with pridopidine (10 and 30 mg/kg) by oral gavage and evaluated for long-term effects (up to 12 months of age) on motor and behavioral disease features.

HD mice showed motor learning deficits, evidenced by a reduced latency to fall during training on the rotarod. These deficits were not observed in mice treated with pridopidine 30 mg/kg. The accelerating rotarod test also identified deficits in motor coordination, which were ameliorated by pridopidine treatment up to 10 months of age. A similar beneficial effect of pridopidine was also seen in the climbing test. The lower dose of 10 mg/kg did not show any beneficial effects [77].

YAC128 HD mice demonstrate anxiety-like behavior, as assessed using the open-field and elevated plus maze, which was reduced in pridopidine-treated mice. Similarly, HD mice exhibited depressive-like behavior in the forced swim test, which was once again alleviated by pridopidine treatment [77]. The lower dose had a diminished effect on anxiety, but a similar effect on depressive-like behavior.

When pridopidine treatment (30 mg/kg) was commenced in mice in later stages of the disease (8 months of age), motor function and anxiety-like behaviors were not affected, but pridopidine did significantly improve depressive-like phenotypes [77]. These data suggest the importance of treatment at early stages of the disease.

An independent study performed in R6/2 HD model mice evaluated the effects of pridopidine (5–6 mg/kg) on motor function and survival. Presymptomatic mice were administered pridopidine intraperitoneally for six weeks, and beneficial effects were observed in both the horizontal-ladder motor test and the open-field test and were maintained for 4 weeks. In the same mice, pridopidine extended lifespan, suggesting a neuroprotective effect further supported by the upregulation of neuroprotective molecules BDNF and dopamine and cAMP regulated phosphoprotein (DARPP)32 in the striatum [146].

Pridopidine’s beneficial effects are further confirmed in vivo in the SOD1 ALS model (mSOD1^G93A^). Mice were treated daily with pridopidine (30 mg/kg), administered subcutaneously for 11 weeks. Mice treated with pridopidine showed a significant ~50% reduction in mSOD1 aggregates, a hallmark of ALS, in both the gray and white matter of the spinal cord. Additionally, in these mice, muscle fiber atrophy of the gastrocnemius muscle was ameliorated, together with a ~50% increase in the number of healthy, innervated NMJs compared to saline-treated mice [79].

In an independent study using a modified mSOD1G93A transgenic mouse, in which disease onset is delayed, the effects of pridopidine (3 mg/kg) administered subcutaneously daily for 4 weeks were evaluated. Even a low dose of pridopidine with a shorter administration (3 mg/kg/day for 4 weeks) was sufficient to ameliorate ALS pathology. Motor coordination was evaluated using a pole test, in which untreated mSOD1 mice took longer to climb down the pole, which was improved by pridopidine treatment. Gait analysis also showed beneficial effects of pridopidine on motor coordination. Additionally, pridopidine prevented cachexia, a key feature of ALS, in this model [75].

## 7. Clinical Evidence

### 7.1. Pridopidine Efficacy in HD

The efficacy of pridopidine was evaluated in four randomized, placebo-controlled trials in adult-onset HD patients. Originally postulated to be a dopamine stabilizer, due to its effects on locomotor function in rodents [147,148], pridopidine was initially developed to treat motor symptoms. Accordingly, the first two trials, HART and MermaiHD, were short-term studies, 12 and 26 weeks, respectively, with the primary endpoint being the modified Motor Score (mMS). This modification of the gold-standard Total Motor Score (TMS) section of the Unified Huntington’s Disease Rating Scale (UHDRS) does not include key features of HDdystonia, chorea, and eye movements [149].

Nevertheless, in both trials pridopidine demonstrated consistent beneficial effects on the TMS. In the Phase 2 HART trial, the 45 mg bid (twice daily) dose showed a nominally significant benefit to TMS vs. placebo (Δ = −2.8, *p* = 0.04), particularly in hand movements (Δ = −0.93, *p* = 0.02) and gait and balance (Δ = −0.48, *p* = 0.04) [150]. In the MermaiHD trial, the 45 mg bid dose showed an effect vs. placebo in the mMS (Δ = −0.99, *p* = 0.042) that did not meet the primary endpoint (the α level was 0.025 for the primary analysis). A greater significant effect was observed on TMS (Δ vs. placebo −2.96, *p* = 0.04). This confirmed results from the HART study, with the most significantly affected items on the TMS being hand movements, gait and balance, together with eye movements and dystonia [151].

These encouraging results informed the design of the PRIDE-HD trial, a Phase 2, multiple-dose, randomized, placebo-controlled trial. PRIDE-HD was originally planned as a 26-week trial with change from baseline in TMS as the primary endpoint. However, midway through the trial, pridopidine’s MOA as an S1R agonist was clarified and elucidated. Given the known neuroprotective properties of S1R activation, this revelation suggested a possible therapeutic benefit for pridopidine beyond motor symptom management, as a possible disease-modifying agent. As a result, the study was extended to 52 weeks, and total functional capacity (TFC) was added as an exploratory endpoint [152].

The primary endpoint of TMS at 26 weeks was not met, but a significant effect was seen on TFC at 52 weeks in the 45 mg bid group [152]. Post hoc analysis revealed a nominally significant effect of pridopidine vs. placebo at 52 weeks on TFC in the entire population (Δ = 0.87, *p* = 0.0032, positive change indicates improvement), which was more pronounced in patients with mild-to-moderate disease (baseline TFC of 7–13) (Δ = 1.16, nominal *p* = 0.0003) [153].

The phase 3 PROOF-HD trial was designed with the data garnered from PRIDE-HD, focusing on the mild-to-moderate HD population and the 45 mg bid dose. The trial evaluated pridopidine’s impact on multiple clinical measures of disease progression, including functional, motor, and cognitive decline, with the double-blind study ending at 65 weeks [154].

Given the long duration of the trial during the COVID-19 pandemic, the use of antidopaminergic medicines (ADMS; i.e., VMAT2i inhibitors, used to treat chorea, and antipsychotics, commonly used to treat behavioral as well as motor symptoms of the disease) was more frequently used than expected [154].

Many ADMs have known side effects, which include sedation and cognitive impairment [155,156,157,158]. These limitations have the potential to interfere with clinical measures and may affect their sensitivity; therefore, analysis of participants not taking ADMs was predefined.

In the subgroup of participants not taking ADMs, pridopidine consistently showed favorable trends compared to placebo across multiple measures of function, cognition, motor performance, and cUHDRS scores. Nominally significant benefits were observed in the cUHDRS up to 52 weeks (vs. placebo: at week 26 Δ = 0.46, *p* = 0.004; week 39 Δ = 0.45, *p* = 0.014; week 52 Δ = 0.41, *p* = 0.035), with favorable trends at weeks 65 (Δ = 0.27 *p* = 0.168). All components of the cUHDRS favored pridopidine vs. placebo [154], including cognition (Stroop Word Reading (SWR) test, vs. placebo: at week 26 Δ = 3.16, *p* = 0.018; week 39 Δ = 2.89, *p* = 0.058; week 52 Δ = 3.05, *p* = 0.042). These results are further supported by the Q-Motor measures, which provide a non-biased, rater- and site-independent assessment of fine motor function. Moreover, the finger tapping inter-onset interval (FT IOI) measure of Q-Motor is a particularly sensitive measure for assessing longitudinal progression, correlating with striatal atrophy and functional decline [159]. In PROOF-HD, pridopidine treatment resulted in improved FT IOI performance, with nominally significant effects at weeks 26 (Δ vs. placebo = −21.15 msec, *p* = 0.025), 65 (Δ = −24.71 msec, *p* = 0.016), and 78 (Δ = −22.90 msec, *p* = 0.028) [154].

### 7.2. Pridopidine Efficacy in ALS

The safety and efficacy of pridopidine 45 mg bid in ALS patients was evaluated in the Phase 2 HEALEY ALS Platform Trial over 24 weeks. At the time of enrollment, broad inclusion criteria were applied and included participants meeting all definitions of El Escorial Criteria (EEC), and in advanced stages of the disease (up to 36 months from disease onset). No differences vs. placebo were observed in the primary endpoint of change from baseline in the ALS Functional Rating Scale—Revised (ALSFRS-R), nor in secondary endpoints or subdomains of the ALSFRS-R. However, predefined subgroup analysis in early, rapidly progressing patients (definite ALS by EEC up to 18 months from symptom onset) suggested slower disease progression in ALSFRS-R Total score (Δ vs. placebo = 3.21, *p* = 0.07) and ALSFRS-R respiratory (Δ vs. placebo = 1.22, *p* = 0.09), especially dyspnea (Δ vs. placebo = 1.33, *p* = 0.001).

These findings, together with the knowledge that previous clinical trials demonstrating efficacy in ALS predominantly enrolled patients with definite or probable ALS by EEC up to 24 months from symptom onset [160,161], informed a post hoc analysis conducted on a more homogenous group of patients with definite or probable ALS up to 18 months from symptom onset [162].

Within this enriched subgroup, pridopidine showed favorable and significant effects vs. placebo, slowing the decline in multiple measures, including disease progression (ALSFRS-R) (Δ vs. placebo 2.9, *p* = 0.03), bulbar (Δ = 0.93, *p* = 0.06), and respiratory function (Δ = 1.2, *p* = 0.03) at 24 weeks [162,163].

Pridopidine’s effects on respiration and bulbar function align with previously reported effects of the non-selective S1R agonist Nuedexta^®^ (20 mg dextromethorphan + 10 mg quinidine) on swallowing and saliva control, further supporting the potential of the S1R as a therapeutic target in ALS [164,165]. Notably, pridopidine showed significant effects on quantitative speech measures, slowing the decline in multiple measures, primarily speaking (Δ vs. placebo = 0.19, *p* = 0.03) and articulation (Δ = 0.21, *p* = 0.01) rates in the full analysis set.

### 7.3. Safety

In all double-blind clinical trials, as well as in open-label extensions, pridopidine demonstrated a safety and tolerability profile comparable to placebo [150,151,152,166,167]. While pridopidine has a concentration-dependent effect on the Fridericia-corrected QT interval, the effect of the therapeutic dose of 45 mg bid was found to be not clinically relevant, and the rate of cardiac-related adverse effects was similar to that of the placebo group [168].

A recent integrated safety efficacy analysis was performed on the pooled safety database of ~1000 HD patients. No new safety signals emerged in this larger cohort, and the analysis replicated and corroborated the placebo-like safety profile observed in the individual studies. Long-term exposure data from open-label extension studies (up to 6.5 years) confirm its safety and tolerability, and support its potential for long-term use [169].

The observed clinical effects may be attributed to the neuroprotective effects of S1R activation. By rescuing the cellular mechanisms that are disrupted in neurodegenerative diseases, pridopidine prevents the degeneration and eventual death of neurons, enabling them to maintain proper function. Moreover, S1R activation by pridopidine increases the availability and signaling of BDNF, which is essential for neuronal survival [170]. Abnormal levels of BDNF and disrupted signaling have been implicated in many diseases of the CNS, highlighting its potential as a transformative target in neurodegenerative diseases [106,171,172,173]. These data support further exploration of the safety and efficacy of pridopidine in HD and ALS patients in Phase 3 clinical trials, focusing on patients early in their disease.

## 8. Potential Future Therapeutic Avenues

Pridopidine’s targeting of the S1R and the cellular mechanisms that are common to several neurodegenerative diseases make it a potential therapeutic for additional diseases and disorders in the nervous system. This potential is supported by preclinical evidence evaluating pridopidine in disease models, but also by evidence from other S1R agonists.

### 8.1. Parkinson’s Disease

In the 6-OHDA mouse model of PD, pridopidine demonstrated neuroprotection of nigral dopaminergic cell bodies, accompanied by reinnervation in the striatum, especially the ventrolateral striatum, which controls forelimb use. Thus, pridopidine administration improved motor capabilities of spontaneous turning behavior, contralateral forelimb use, and gait. In line with the biphasic response, a lower dose of pridopidine showed a greater effect [76]. In this model, pridopidine also demonstrated a reduction in microglial activation, suggesting its potential for modulating neuroinflammation, a key feature of neurodegenerative diseases [76]. These data highlight pridopidine as a promising non-dopaminergic approach to the treatment of PD [174].

### 8.2. Alzheimer’s Disease

The cognitive decline characteristic of AD is the result of synaptic dysfunction and the loss of mushroom spines in the dendrites of hippocampal neurons. As mentioned above, pridopidine prevented the loss of mushroom spines in mouse models of AD and regulated the ER Ca^2+^ flux necessary for spine stability [83]. In older APP/PS1 AD model mice, cognitive impairments affect spatial learning and memory, a phenotype that is rescued by pridopidine [74].

### 8.3. Retinal Degeneration

A large body of evidence points to the S1R as a promising target for treating retinal cell dysfunction and degeneration. Several cellular pathologies are ameliorated by S1R treatment, including mitochondrial dysfunction, oxidative stress, and BDNF signaling [175]. In two different models of retinal degeneration, orally administered pridopidine protected retinal ganglion cells and their axons from cell death due to the elevation of intraocular pressure (IOP). Interestingly, pridopidine’s protective effect is not mediated by alleviating IOP, supporting its potential for treating a variety of retinal degenerations and optic neuropathies [176].

One of the underlying causes of increased IOP is fibrosis, which can hinder the outflow of aqueous humor. S1R activation has been shown to ameliorate the fibrotic response in optic models [177], suggesting that pridopidine treatment may have the potential to modulate IOP as well, offering a two-prong approach to glaucoma treatment.

### 8.4. Neurodevelopmental Disorders

Neurodevelopmental disorders, such as Fragile X Syndrome (FXS) and Rett Syndrome (RTT), are also rare diseases for which there is an urgent need for treatments [178]. Despite their etiological differences, the two share several clinical and pathophysiological phenotypes, which can be explained by the interplay and common targets between FMRP and MeCP2, the proteins in which mutations are causative for FXS and RTT, respectively [62,179]. Overlapping symptoms include intellectual disability, seizures, communication deficits, and skeletal defects. Mitochondrial function and BDNF signaling are disrupted at the cellular level in both diseases, and synaptic function and plasticity are impaired [179].

These shared cellular deficiencies provide the rationale for treating RTT and FXS with S1R agonists. Indeed, the non-specific S1R agonist ANAVEX2-73 (blarcamesine) increased BDNF levels and improved behavioral phenotypes in the FMR1 KO2 mouse model of FXS [180]. In the RTT mouse model MECP2^null (het)^, S1R activation with ANAVEX2-73 improved motor coordination and balance, sensory responses, and respiratory symptoms.

### 8.5. Vanishing White Matter (VWM) Disease

Vanishing White Matter is a rare, genetic, autosomal recessive leukodystrophy characterized by the progressive loss of white matter in the brain. As a result of white matter loss, axons degenerate, leading to the progressive deterioration of neurologic function, paralysis, and eventually, death.

VWM is caused by mutations in any of the five genes encoding the subunits of the eukaryotic initiation factor 2B (*eIF2B*), a master regulator of mRNA translation key to the integrated stress response (ISR), which is activated as part of the UPR. The S1R was identified as a therapeutic target for VWM in a computational screen. This was confirmed by evaluating the effects of pridopidine on primary cells isolated from Eif2b5R^132H/R132H^ cells [181]. Pridopidine rescued mitochondrial membrane potential and ATP production, and enhanced the ability of mutant cells to mitigate chronic ER stress. In the 2b4^he^2b5^ho^ VWM mouse model, pridopidine ameliorated ataxia and measures of gait and balance without affecting the ISR [182].

### 8.6. Wolfram Syndrome (WFS)

Wolfram Syndrome is a severe, rare, autosomal recessive disease with a complex pathology, characterized by diabetes, optic atrophy, and deafness. It is caused by mutations in the *WFS1* gene, which encodes Wolframin, a transmembrane ER protein expressed primarily in spiral ganglion neurons, retinal ganglion cells, and β cells in the pancreas.

In line with Wolframin’s role in Ca^2+^ homeostasis, *WFS1* deficiency alters ER–mitochondria communication at the MAM, causing mitochondrial dysfunction. The S1R’s known functions at the MAM suggest its potential as a therapeutic target for WFS. Activation of the S1R with the non-clinical agonist PRE-084 restored Ca^2+^ homeostasis, mitochondrial function and autophagy in patient-derived cells, and attenuated memory deficits and anxiety in the WFS1^DExon8^ mouse model of the disease. These results encourage the evaluation of pridopidine for the treatment of WFS [183].

## 9. Conclusions

The S1R’s ability to tip the balance between cell death and survival in favor of survival is particularly important in neuronal cells, which are more sensitive to stress and environmental insult. By enabling neurons to preserve proper function, thus delaying the progression of neurodegeneration, S1R activation has the potential to preserve quality of life, reduce disease burden, and delay disability.

Pridopidine stands out as the most advanced and selective S1R agonist in clinical development. It has shown consistent benefits on multiple clinical measures of disease progression in both HD and ALS patients, encompassing functional, cognitive, and motor domains. In both diseases, pridopidine modifies the clinical course of the disease. These findings underscore the central role of S1R activation in promoting cellular resilience and delaying neurodegeneration, with pridopidine emerging as a leading candidate. Additionally, pridopidine’s established safety and tolerability profile, together with its administration as an oral tablet, makes it easy to tolerate and administer. The expanding mechanistic insights into S1R signaling and its relevance across diverse pathologies within the CNS invite further investigation into the therapeutic scope of both S1R activation and pridopidine as an S1R agonist. Non-clinical evidence supports pridopidine’s therapeutic potential in other neurodegenerative diseases, such as retinal degeneration, AD, and PD.

## Figures and Tables

**Table 1:** S1R selectivity of investigational and approved drugs.

Family	Compound	Ki for S1R	S1R Activity	Ki for Other Target	Fold Selectivity for S1R
S1R agonist in development	Pridopidine	57 nM	agonist	ADRα2; 1600 nM	28
	Blarcamesine	3700 nM	agonist	M1; 500 nM	0.14
S1R agonist/ NMDAR antagonist	Dextromethorphan	~100 nM	agonist	NMDAR; 500 nM	5
Cholinesterase inhibitor	Donepezil	14.6 nM	agonist	AChEi; 5.7 nM	0.4
Antipsychotic	Chlorpromazine	180 nM	antagonist	D1R, 56 nM	0.3
	Haloperidol	3.7 nM	antagonist	D2R; 0.89 nM	0.24
Antidepressants	Imipramine	~500 nM	agonist	SERT, 1–3 nM	<0.05
	Fluoxetine	240 nM	agonist		
	Citalopram	292 nM	agonist		
	Fluvoxamine	36 nM	agonist		
	Sertraline	57 nM	antagonist		

## Data Availability

All data included in this review has been previously published and cited accordingly. Data supporting the findings of this manuscript are derived from previously published studies and/or internal clinical trial datasets. No new datasets were generated or analyzed specifically for this work. Access to individual-level data is restricted due to privacy, ethical, and regulatory considerations.
